# *Candida auris* is emerging as a prevalent urinary pathogen

**DOI:** 10.1371/journal.ppat.1013138

**Published:** 2025-05-07

**Authors:** Alyssa Ann La Bella, Felipe Hiram Santiago-Tirado, Ana Lidia Flores-Mireles

**Affiliations:** Department of Biological Sciences, University of Notre Dame, Notre Dame, Indiana, United States of America; University of Maryland, Baltimore, UNITED STATES OF AMERICA

## Abstract

Urinary tract infections (UTIs) are one of the most common infections, with a subgroup of these infections, catheter-associated UTIs (CAUTIs), accounting for 40% of nosocomial infections. While the majority of CAUTI pathogens are bacterial, the second most common pathogen is the fungus *Candida albicans.* However, in recent years, *Candida auris* has increasingly been isolated from urine, indicating *C. auris’* potential as a urinary pathogen. *C. auris* has rapidly emerged as a human pathogen worldwide, becoming a serious health threat. This is of great concern due to its antifungal resistance, adherence to inanimate surfaces, high mortality rates, and the extensive knowledge gap regarding *C. auris’* prevalence and pathophysiology. To understand whether *C. auris* is prevalent in the urinary tract, we analyzed 12,996 *C. auris* clinical strains and their frequency related to urine and urinary catheters. We identified urine as the second most common *C. auris* isolation source in the United States and the third most common worldwide. Anecdotally, *C. auris* urine isolates are often associated with urinary catheters and high mortality rates. Furthermore, there has been an early indication of urinary isolates developing echinocandin resistance. With the increasing incidence of uropathogenic *C. auris*, it is critical to have an in-depth understanding of *C. auris* pathogenesis in the urinary tract to effectively prevent and treat these infections.

## Introduction

*Candida auris* was an environmental fungus until 2009, when it was isolated from an ear infection in Japan [1]. Following that, *C. auris* was recognized as a common cause of severe infection with high mortality in healthcare settings throughout the world [1]. *C. auris’* success is attributed to its multi-antifungal resistance and biofilm formation, aggregation, immune evasion, and thermotolerance, allowing it to survive on inanimate surfaces as well as in human hosts [1]. While the ear canal was the first patient isolation source, *C. auris* has been isolated from other body sites, including axilla, urine, bloodstream, and skin.

Notably, urine has become a predominant *C. auris* isolation source in patients, emerging as a urinary pathogen (uropathogen) [2]. Urinary tract infections (UTIs) are common infections classified as uncomplicated or complicated [3]. Uncomplicated UTIs (uUTIs) occur in healthy individuals without pre-existing conditions by uropathogenic *Escherichia coli* [3]. Conversely, complicated UTIs (cUTIs) are associated with urinary obstruction/structural abnormality, a compromised urinary tract, or underlying medical conditions. Examples include patients with urinary catheters, kidney stones, renal failure, transplantation, and obstructions [3].

Despite its benefits, urinary catheterization renders patients susceptible to catheter-associated UTIs (CAUTIs), accounting for 80% of cUTIs and ~ 40% of all hospital-acquired infections [4]. Unlike uUTIs, CAUTIs are not gender or age-specific and are caused by both bacteria and fungi [3]. It is estimated that ~15–25% of hospitalized patients require a urinary catheter, with use increasing to 60% in intensive care unit (ICU) patients [5]. In nursing homes, ~ 7% of residents worldwide [6] and ~ 11.9% of residents in the United States (US) utilize catheters [7], and ~ 50% will experience symptomatic CAUTIs [8]. Persistent CAUTIs often lead to urosepsis, septicemia, and death [3]. Importantly, recent clinical studies indicate that CAUTI is an independent and significant risk factor for ICU mortality [9].

Recently, *Candida* species, specifically *C. albicans*, have increased their prevalence in the urinary tract, accounting for 17.8% of CAUTIs [3]. Yet fungal CAUTIs remain understudied compared to its bacterial counterparts [3,10]. Alarmingly, 25% of sepsis cases are from urinary isolates [11]; thus, understanding fungal infections is critical to reduce patient urosepsis, candidemia, and mortality [12].

Importantly, with *C. auris* outbreaks in healthcare facilities and fungal UTI/CAUTI increase, it is essential to investigate *C. auris* as a uropathogen. Here, we analyze *C. auris* clinical data in relation to the urine environment and as a uropathogen.

### Where is *C. auris* isolated from?

We identified and quantified the various isolation sources of *C. auris* clinical isolates worldwide (>12,000) from NCBI’s Pathogen Database into the following 13 categories: axilla/groin/nares; urine/urinary catheter (urinary); blood; skin; wound; respiratory tract (respiratory); sputum; ear; tissue (i.e., various bones and tissue types); unspecified catheter; non-urinary catheter (i.e., central venous/blood lines, dialysis catheters, nephrostomy tubes, and lumbar punctures); other; and unknown/not stated (unknown).

**Fig 1:**
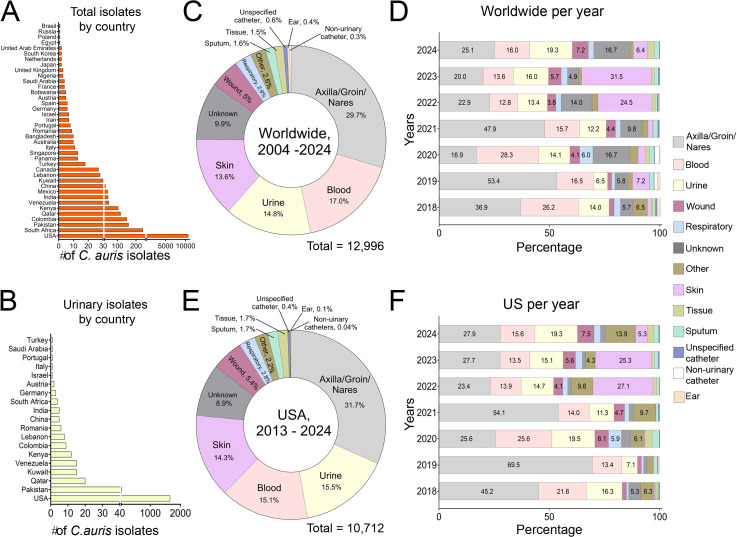
*Candida auris* prevalence and isolation sources. (**A**) The number of *C. auris* isolates by country according to the NCBI Pathogen Database. (**B**) The number of *C. auris* urinary isolates by country. (**C**) Composition of *C. auris* isolates worldwide from 2004 to 2024. (**D**) Yearly breakdown, from 2018 to 2024, of *C. auris* isolation sources worldwide. (**E**) Composition of *C. auris* isolates in the US from 2013 to 2024. (**F**) Yearly breakdown, from 2018 to 2024, of *C. auris* isolation sources in the US.

*C. auris* clinical isolates (*n* = 12,996) originated from 39 different countries (Fig 1A) with urinary isolates present in 19 countries (Fig 1B). Worldwide data (2004–2024) show that axilla/groin/nares were the most prevalent isolation source, comprising 29.7% of isolates (Fig 1C). Blood (17.0%), urine/urinary catheter (14.8%), and skin (13.6%) were the following most common sources (Fig 1C). A yearly breakdown (2018–2024) showed that axilla/groin/nares isolates were particularly abundant in 2019 (53.4%) and 2021 (47.9%) (Fig 1D). Interestingly, worldwide, urine/urinary catheter isolates increased from 2022 to 2024, becoming the second most common isolation source (19.3%) in 2024 (Fig 1D).

A large proportion (~80%) of *C*. *auris* clinical strains were isolated in the US, followed by South Africa and Pakistan (Fig 1A). Similarly, majority of the urinary isolates were from the US, followed by Pakistan and Qatar (Fig 1B). From 2013 to 2024, 10,712 isolates have been reported, where31.3% were axilla/groin/nares isolates, the most common isolation source (Fig 1E). Urine/urinary catheters are the second most common isolation source (15.5%), followed by blood (15.1%) and skin (14.3%) (Fig 1E). A 2018–2024 yearly analysis of *C. auris* isolates in the US showed that axilla/groin/nares isolates are the most prevalent each year (Fig 1F). Notably, from 2021 to 2024, urine/urinary catheter isolates increased from 11.3% to 19.3%, with the highest prevalence in 2020. This sudden increase in urinary isolates may be attributed to the hospitalization increase in 2020 after the COVID-19 pandemic. Concerningly, the 2024 urinary isolate prevalence (19.3%) is approaching 2020 (19.5%).

*C. auris* cases may be underestimated in this study since it relies on isolates/cases reported to the NCBI database. Another hurdle was the lack of detailed information about the source site. For example, some reports lacked the specific site of fungal isolation, rather clustering isolation sources (axilla/groin/nares) instead of providing a single source (blood). Additionally, other reports lacked information on catheter type or tissue where samples were isolated. Finally, some isolates had multiple isolation sources (urine and blood), creating ambiguity about the pathogen’s origin.

### Urinary isolates: uUTI or CAUTI?

Often, when the isolation source was listed as “urine”, it remained unspecified whether patients had an indwelling urinary catheter, making it difficult to associate *C. auris* with uUTI or CAUTIs. To understand if *C. auris* urine isolates coincide with urinary catheter usage, we examined five case studies that provided additional patient details:

1. A 2024 study from rural West India found that >60% (6/9) of *C. auris* cases were urinary isolates. Of those six patients, five isolates came from urine while one was isolated from a urinary catheter. Furthermore, all five patients with a urine isolate had an indwelling catheter. Five patients died and one patient left against medical advice [13].
2. A 2024 study from a Bahrain tertiary care hospital analyzed 59 patient samples with *C. auris* invasive infection or colonization. The most prevalent isolation sources were groin, with 20 samples (33.9%), and urine, with 15 samples (25.4%). Twenty-three patients had a urinary catheter (44.2%); although it is unknown whether they had a corresponding urine isolate. Twenty-six patients died (44.1%), wherefrom seven patients (26.9%) had urine isolates [14].
3. A 2023 study in Brazil found that 2 of 11 *C. auris* isolates originated from urine. Both patients had an indwelling urinary catheter and succumbed to their illnesses [15].
4. In a 2023 Saudi Arabia study, *C. auris* was isolated from 53 patients, with 16 isolates from urine (30.2%). Of the 53 patients, 17 (32.1%) had an indwelling medical device; however, which patients and the medical device type were not detailed [16].
5. During a 2020 COVID-19 outbreak in a Lebanese tertiary care facility, 14 *C. auris* cases were documented. Ten samples came from the respiratory tract (71.4%) and three from urine (21.4%). Two patients with urinary isolates had indwelling urinary catheters. The urinary isolate patient without the urinary catheter died of septic shock [17].

While we cannot directly associate *C. auris* urine isolates with urinary catheter usage, the anecdotal evidence from the case reports suggests that these isolates frequently coincide with urinary catheters. Studies that identify/correlate positive urine samples with urinary catheter presence are needed to understand if *C. auris* causes CAUTIs, uUTIs, or both.

### Are *C. auris* uropathogens developing multidrug resistance?

An important trend regarding urinary isolate-antifungal resistance is noted. While most *C. auris* isolates have developed resistance to azoles, *C. auris* echinocandin (caspofungin, micafungin, and anidulafungin) resistance is less frequent [1]. While datasets on multidrug resistance for specific isolation sources are not currently compiled or available, several studies have found echinocandin resistance in *C. auris* urine isolates. A study described that a patient had *C. auris* isolates from trachea secretion and urine. The trachea isolate remained echinocandin-susceptible, but the urinary isolate developed resistance [18]. A 2018–2019 study in Kuwait identified three *C. auris* urinary isolates with reduced echinocandin susceptibility [19]. A 2018–2021 United Arab Emirates study found urinary isolates had the highest echinocandin resistance, with 9.3% and 4.2% developing resistance to caspofungin and micafungin, respectively [20]. Finally, a US case study showed a 54-year-old male with an indwelling urinary catheter developed echinocandin-resistant candidemia after several positive *C. auris* urine cultures [21]. The fact that *C. auris* urine isolates exhibited higher echinocandin resistance is unsurprising since the majority of uropathogens are developing multi-drug resistance [3]. Multi-drug resistance can be attributed to biofilm formation but also could be related to antimicrobial concentration in the bladder, which is affected by constant urine accumulation and voiding.

Effective treatment of urinary infections with echinocandins is challenged by their limited urinary tract concentrations, despite higher levels in blood and sputum [22]. Suboptimal echinocandin concentrations during repeated treatment of urinary *C. auris* infections could drive the evolution of resistance in urinary isolates [22,23]. While echinocandins remain effective against *C. auris* isolates from ear [24] and bloodstream [25] infections, urinary isolates exhibit altered antifungal susceptibility [18–21]. These findings warrant future studies about *C. auris* urine isolates and their ability to develop resistance to these last resort antifungals.

### Why is the catheterized bladder ideal for *C. auris*?

A healthy bladder maintains an environment hostile to pathogens through frequent urine voiding and a urine composition that is naturally hypertonic, acidic, rich in urea, and contains antimicrobial agents [26,27]. As the most prevalent urinary tract pathogen, uropathogenic *E. coli* has adapted to thrive in a healthy bladder by acquiring nutrients and employing immune evasion mechanisms, notably host cell invasion and intracellular bacterial community formation [3]. However, urinary catheterization alters the bladder environment, promoting infection by pathogens that typically do not infect healthy bladders. This is due to the physical bladder trauma caused by the catheterization, resulting in inflammation and recruitment of serum proteins, including fibrin(ogen), a blood coagulation factor, to facilitate tissue repair [3]. Fibrin(ogen) deposition on the bladder and catheter becomes a scaffold for microbial biofilms and is critical for CAUTIs caused by gram-negative, gram-positive, and fungal pathogens including *C. albicans* [3,10]. Furthermore, serum extravasation provides nutrients for these opportunistic pathogens [3]. Thus, the catheterized bladder environment provides numerous niches and nutrients that can be exploited by *C. auris.* Furthermore, *C. auris*’ ability to form biofilms on materials like silicone, latex, and plastic [1], commonly used in urinary catheters, allows for its attachment during placement, promoting bladder infection. *C. auris’* adherence capability facilitates its spread throughout healthcare settings via contaminated gloves, bedrails, and medical equipment [1], posing a significant threat.

## Conclusion

**Fig 2:**
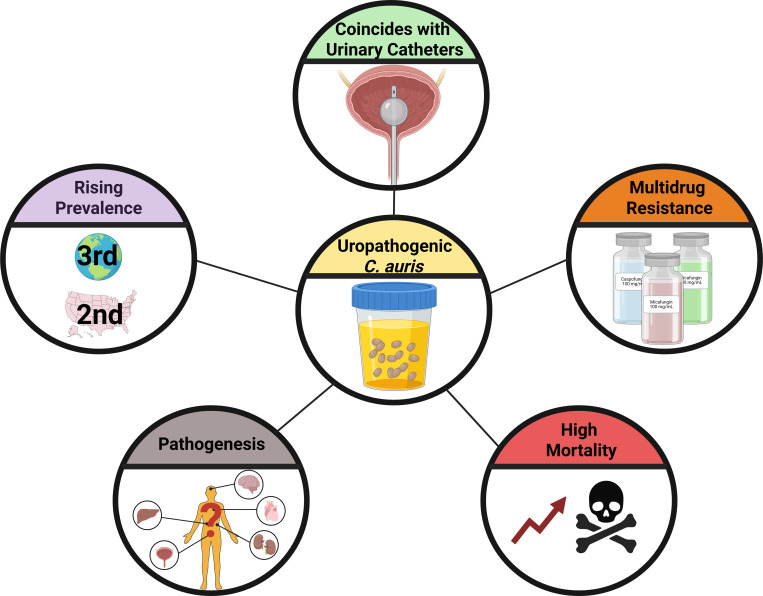
Summary of *Candida auris* as a uropathogen. From database and literature analysis, this study shows that uropathogenic *C. auris* isolates are (1) rising as a prevalent source, (2) coinciding with urinary catheter usage, (3) developing multidrug resistances, (4) have high mortality, and (5) have unknown pathogenesis mechanisms. Created in Biorender [28].

In summary, *C. auris* is increasingly isolated from urine, becoming the third most common isolation source worldwide (14.8%) and the second most prevalent in the US (15.5%). Clinical reports showed a large proportion of these urine isolates coincide with urinary catheters and high mortality (Fig 2). This suggests that *C. auris* UTIs/CAUTIs should not be ignored, especially if patients have comorbidities that could contribute to disseminated infection. Clinical studies also revealed that urine isolates are developing echinocandin-resistance (Fig 2). Despite *C. auris’* prevalence in the urinary tract, we lack fundamental knowledge about it as a uropathogen. As urinary catheters, and subsequently CAUTIs, become more prevalent, it is essential to dissect how this superbug causes infection to develop efficient intervention strategies to improve infection outcome for patients.
